# Identifying Reddit Users at a High Risk of Suicide and Their Linguistic Features During the COVID-19 Pandemic: Growth-Based Trajectory Model

**DOI:** 10.2196/48907

**Published:** 2024-08-08

**Authors:** Yifei Yan, Jun Li, Xingyun Liu, Qing Li, Nancy Xiaonan Yu

**Affiliations:** 1 Department of Social and Behavioural Sciences City University of Hong Kong Hong Kong China (Hong Kong); 2 Department of Computing The Hong Kong Polytechnic University Hong Kong China (Hong Kong); 3 Key Laboratory of Adolescent Cyberpsychology and Behavior Central China Normal University Ministry of Education, School of Psychology Wuhan China

**Keywords:** COVID-19 pandemic, Reddit, suicide risk, trajectory

## Abstract

**Background:**

Suicide has emerged as a critical public health concern during the COVID-19 pandemic. With social distancing measures in place, social media has become a significant platform for individuals expressing suicidal thoughts and behaviors. However, existing studies on suicide using social media data often overlook the diversity among users and the temporal dynamics of suicide risk.

**Objective:**

By examining the variations in post volume trajectories among users on the r/SuicideWatch subreddit during the COVID-19 pandemic, this study aims to investigate the heterogeneous patterns of change in suicide risk to help identify social media users at high risk of suicide. We also characterized their linguistic features before and during the pandemic.

**Methods:**

We collected and analyzed post data every 6 months from March 2019 to August 2022 for users on the r/SuicideWatch subreddit (N=6163). A growth-based trajectory model was then used to investigate the trajectories of post volume to identify patterns of change in suicide risk during the pandemic. Trends in linguistic features within posts were also charted and compared, and linguistic markers were identified across the trajectory groups using regression analysis.

**Results:**

We identified 2 distinct trajectories of post volume among r/SuicideWatch subreddit users. A small proportion of users (744/6163, 12.07%) was labeled as having a high risk of suicide, showing a sharp and lasting increase in post volume during the pandemic. By contrast, most users (5419/6163, 87.93%) were categorized as being at low risk of suicide, with a consistently low and mild increase in post volume during the pandemic. In terms of the frequency of most linguistic features, both groups showed increases at the initial stage of the pandemic. Subsequently, the rising trend continued in the high-risk group before declining, while the low-risk group showed an immediate decrease. One year after the pandemic outbreak, the 2 groups exhibited differences in their use of words related to the categories of personal pronouns; affective, social, cognitive, and biological processes; drives; relativity; time orientations; and personal concerns. In particular, the high-risk group was discriminant in using words related to anger (odds ratio [OR] 3.23, *P*<.001), sadness (OR 3.23, *P*<.001), health (OR 2.56, *P*=.005), achievement (OR 1.67, *P*=.049), motion (OR 4.17, *P*<.001), future focus (OR 2.86, *P*<.001), and death (OR 4.35, *P*<.001) during this stage.

**Conclusions:**

Based on the 2 identified trajectories of post volume during the pandemic, this study divided users on the r/SuicideWatch subreddit into suicide high- and low-risk groups. Our findings indicated heterogeneous patterns of change in suicide risk in response to the pandemic. The high-risk group also demonstrated distinct linguistic features. We recommend conducting real-time surveillance of suicide risk using social media data during future public health crises to provide timely support to individuals at potentially high risk of suicide.

## Introduction

The COVID-19 pandemic has triggered a global increase in mental disorders [1], with heightened concerns about suicide risk both in the short and in the long term [2,3]. While an interrupted time-series study indicated that most countries or regions have not experienced a significant rise in suicide deaths [4], the prevalence of suicidal ideation (10.81%-12.10%) and suicide attempts (4.86%) has notably increased compared with prepandemic levels [5,6]. Our recent meta-analysis, utilizing longitudinal data, has further corroborated an increase in the prevalence of suicidal ideation and suicide attempts among both nonclinical and clinical populations following the onset of the pandemic [7]. Suicide remains a critical public health concern throughout the pandemic, underscoring the need for ongoing monitoring and vigilance as the situation continues to evolve [8,9]. In this study, we utilized posts from a social media forum to examine patterns of change in suicide risk during the COVID-19 pandemic, analyzing their corresponding linguistic features.

Social media and online discussion forums are increasingly recognized as valuable resources for suicide research [10,11]. These virtual platforms offer users open and widely accessible spaces to share experiences, engage in discussions, seek social support, and exchange information anonymously and in real time [12,13]. Compared with traditional clinical data sets, these online data sets offer several advantages: they are publicly and freely available, involve larger sample sizes, provide access to participants who are ordinarily difficult to engage, enable comparisons with historical data, and exhibit high ecological validity through documentation of first-person experiences [14]. Utilizing social media data from platforms such as Twitter/X (X Corp.), Reddit (Reddit, Inc.), and Weibo (Weibo Corporation), previous studies have detected and predicted suicide risk and developed intervention programs [15-21]. As a result of lockdown measures and heightened concerns during the pandemic, social media data have become even more valuable for suicide research, as these online platforms have become the primary means for many people to receive information and stay connected with the outside world [22,23].

The r/SuicideWatch subreddit is a semianonymous forum that provides “peer support for anyone struggling with suicidal thoughts or worried about someone who may be at risk” [24]. This makes it an important resource for suicide research, as it offers high-quality, self-reported suicide data. Dominant suicide risk assessments for online posts, such as machine learning models combined with manual coding, typically approach it as a multiclassification task. These models output the post-level suicide risk (ie, the risk associated with a single post) using ordinal data. For example, they might label a post as being at ideation, behavior, or attempt level based on the probability score for each level [25-27]. However, such data cannot capture user-level suicide risk (ie, the risk of a user based on posts during a specific period) and may not conform to the distributional assumptions of many statistical analyses, such as growth-based trajectory models, potentially introducing biases [28,29]. To assess users’ overall risk and track temporal changes in risk, researchers can consider both the quantity and quality of posts published, including methods such as topic modeling or linguistic analysis of content [30,31]. Specifically, the quantitative method focuses on the total number of posts (ie, post volume) within a specific period, which indicates users’ posting activity and social engagement level [32,33]. Changes in users’ post volume can reflect changes in their suicide risk. For instance, the diurnal and weekly patterns of post volume on r/SuicideWatch corresponded to temporal fluctuations in suicide risk, including actual suicide attempts or deaths [34]. Similarly, Twitter users exhibited an increased volume of suicide-related posts before their suicide attempts [35]. Furthermore, analyses of both the quantity and quality of posts have demonstrated that higher post volume (quantity) corresponds to active disclosure of suicidal thoughts in post content (quality) [30,31]. Therefore, post volume in online suicide communities can serve as an effective indicator of user-level suicide risk, offering sufficient accessibility and flexibility for statistical analysis.

In this study, we monitored users’ post volume on the r/SuicideWatch subreddit before and during the pandemic to observe changes in their suicide risk. Among active adolescent users during the pandemic (April 2020-September 2021), post volume remained stable compared with prepandemic periods [36]. However, when examining post volume over shorter intervals, it fluctuated and demonstrated an overall decrease until December 2020 [37,38]. There is currently no documented study on how post volume has evolved beyond 2020. It is important to note that users of r/SuicideWatch may vary in their levels of suicide risk, and the findings mentioned above could be ambiguous without accounting for this heterogeneity among users. Through expert annotation, active r/SuicideWatch users (ie, those with at least 10 total posts) were categorized into 4 risk levels: no risk (36/245, 15%), low risk (50/245, 20%), moderate risk (115/245, 47%), and severe risk (44/245, 18%) of suicide [39]. However, each user’s risk label was determined based on the highest risk observed in their posts during a 7-year period before the pandemic. As suggested by the fluid vulnerability theory [40], individual suicide risk is best understood as a temporal process influenced by both baseline and acute risk factors. Environmental stressors or contexts such as sudden outbreaks of infectious disease epidemics or pandemics, social isolation, and fear [3,7] can easily trigger acute suicide risk in individuals predisposed to underlying vulnerabilities (ie, those with a higher baseline risk). Cognitive, emotional, behavioral, and physiological factors interact to either sustain or alleviate their suicide risk [41]. Considering both stability and dynamism, some individuals exhibit fluctuations in suicide risk, moving between low and high states where suicidal behavior may be more or less likely to emerge, while others display a stable pattern. To address the heterogeneity among users and understand the temporal nature of suicide risk, using trajectory modeling techniques such as group-based trajectory modeling (GBTM) and growth curve modeling can be beneficial. These methods identify subgroups within a population that share similarities in outcomes over time [42].

The linguistic or language styles used in posts or comments can offer valuable insights into the experiences and perspectives of suicidal individuals. This can assist researchers in understanding the underlying thoughts, emotions, and behaviors that individuals may be unwilling or unable to express explicitly [43]. Importantly, linguistic features can act as markers that distinguish suicide-related posts from general posts, aiding in the identification of potential high-risk users who may need support [17,44,45]. Suicide-related social media posts often exhibit characteristics such as simplicity in words and short sentences, reduced lexical diversity, and language disorganization [46]. They may also include more statements related to self-destruction, commands, and conflicts [47], along with increased use of first-person pronouns, adverbs, and multifunctional words. These posts frequently reference death, anger, and the present moment, while showing fewer occurrences of second- and third-person pronouns, nouns, and references to causes and differentiation [17,48,49]. However, it remains unclear whether high-risk users have exhibited these linguistic features during the COVID-19 pandemic, which has introduced different stressors potentially influencing suicide risk. Existing studies during the pandemic that utilized psycholinguistic analysis of r/SuicideWatch posts have primarily concentrated on monitoring temporal shifts in these linguistic features. Specifically, studies have identified increased use of words associated with negative emotions and a focus on the past, along with fewer references to positive emotions, social interactions, and leisure activities. References to death and first-person pronouns remained stable [36,37]. However, these findings only encompassed a limited time frame during the pandemic (until September 2021) and did not account for the heterogeneity among users in terms of suicide risk. Different users may exhibit varying linguistic characteristics and trends in linguistic changes over time. Therefore, it is crucial to first identify groups of users exhibiting similar patterns of suicidal behavior throughout the pandemic. Subsequently, analyzing their respective linguistic trends and markers can yield valuable insights for suicide surveillance and targeted interventions among high-risk users.

Utilizing posts from the r/SuicideWatch subreddit before and during the COVID-19 pandemic (March 2019-September 2022), this study aimed to investigate the following: (1) the potential for distinct patterns of change in suicide risk using GBTM of users’ post volumes, (2) the trends and characteristics of linguistic features within posts across each trajectory group, and (3) the linguistic markers associated with each trajectory group. We anticipated that identifying trajectories of post volume on the r/SuicideWatch subreddit would uncover users’ diversity by accounting for the temporal dynamics of suicide risk. Analyzing their associated linguistic features could also help identify users potentially at high risk of suicide. These findings could have significant implications for enhancing suicide screening, monitoring, and interventions during future public health crises.

## Methods

### Data Set and Participants

For this study, we gathered the longitudinal data set using the Reddit application programming interface [50]. Following a previously established method [14], we crawled posts from users who contributed to the r/SuicideWatch subreddit between March 1, 2020, and August 31, 2022, resulting in a total of 603,802 posts from 6943 users. We expanded our data set by retrieving historical posts dating back to March 1, 2019, to analyze the trajectory of post volume before and during the pandemic. To streamline data usage in subsequent analyses, we excluded accounts that were canceled and posts with deleted content. The final data set comprised 6163 users and their posts from the r/SuicideWatch subreddit (N=33,714) spanning the period from March 1, 2019, to August 31, 2022, encompassing the COVID-19 pandemic period. As a result of the onset of the COVID-19 pandemic around March 2020, there was a notable increase in discussions related to the pandemic on Reddit [37]. Therefore, we used March 1, 2020, as a cutoff point and defined 2 prepandemic periods (T1: March 1, 2019-August 31, 2019 and T2: September 1, 2019-February 29, 2020) and 5 peripandemic periods (T3: March 1, 2020-August 31, 2020; T4: September 1, 2020-February 28, 2021; T5: March 1, 2021-August 31, 2021; T6: September 1, 2021-February 28, 2022; and T7: March 1, 2022-August 31, 2022) to track post volume trajectories across these time frames.

### Trajectory Variable: Post Volume

To assess changes in suicide risk, we used post volume from the r/SuicideWatch subreddit within each pre- and peripandemic period for each user as a proxy for the trajectory variable [30,31,34,35,51]. We quantified the number of posts made by each user during each specific period, with periods where no posts were made recorded as 0. For users who joined r/SuicideWatch after March 1, 2020 (ie, those who began posting suicide-related content on r/SuicideWatch only after the pandemic outbreak; 5759/6163, 93.44%) [48], their post counts on r/SuicideWatch during the 2 prepandemic periods and the peripandemic periods before their initial post were recorded as 0. As noted by De Choudhury et al [48], there are a few suicide-related posts found on subreddits outside of r/SuicideWatch. The transition of these users to r/SuicideWatch may indicate the onset and progression of their suicidal concerns following the pandemic outbreak. Therefore, their 0 post volume during the pre- and peripandemic periods can serve as a proxy for their respective suicide risk trajectories, reflecting changes in their behavioral patterns and suicide risk following the pandemic. According to our eligibility criteria, all users included in the study had posted at least once across all periods.

There might be a concern that some of our collected posts discussed the suicide risk of others rather than the user’s own risk, although previous studies have indicated that posts on r/SuicideWatch primarily focus on self-directed concerns [30,48]. To address this issue in our data set, we randomly selected 10% (3372/33,714) of the total posts and manually screened the content to determine the subject of the posts. Among the 3372 posts, only 18 (0.53%) were found not to be about the user’s own suicide-related issues: 6 discussed others’ suicide risk, 5 provided help to others, 3 were about irrelevant topics, and 4 were unclassified. Thus, the majority of our collected posts accurately reflected users’ own experiences and concerns related to suicide risk.

### Linguistic Features

To analyze the linguistic features of posts, we used Linguistic Inquiry and Word Count (LIWC) 2015 [52], a widely used tool for language analysis. LIWC encompasses more than 80 word categories, each containing hundreds of dictionary words for the identification and analysis of word use patterns related to suicide risk. The primary psycholinguistic categories in LIWC are personal pronouns and words related to affective, social, cognitive, perceptual, and biological processes; drives; time orientations; relativity; and personal concerns. For each Reddit user included in our study, we calculated LIWC measures for these 10 major psycholinguistic categories during each period [53]. First, we tallied the occurrences of each word within a specific post alongside the post’s length (ie, the total number of words used). Second, we summed the occurrences of each word and the total length of posts for each period. Finally, for each period, we calculated the normalized frequency of word use in each LIWC category by dividing the total count of the LIWC category by the total length of posts in that period. Therefore, each LIWC measure represents the normalized frequency of word use within a specific LIWC category during each period analyzed.

### Statistical Analysis

GBTM was used to define trajectory groups based on post volume across the COVID-19 pandemic. As a finite mixture model, GBTM is capable of identifying distinct groups of individuals with similar developmental trajectories in a particular outcome or behavior within a population. It accommodates trajectory variables that adhere to distributions such as censored normal (CNORM), zero-inflated Poisson (ZIP), beta, and Bernoulli distributions [54]. Within each period, the exploratory analysis revealed that a large number of eligible users had 0 posts, resulting in a skewed and zero-inflated distribution of the trajectory variable (ie, post volume within each period). Among the available models, the ZIP model was selected to fit our data because of its capability to address excessive zeros. The ZIP model combines an inflation model for zeros with a count model for nonzero values, making it suitable for our data set [54,55].

To identify the best-fitting model with the optimal number of trajectory groups, we followed 3 steps [56]. Initially, we incrementally increased the number of group specifications from 2 to 5 to pinpoint the optimal number of trajectories. Specifically, we selected the model based on 4 commonly used fit statistics in GBTM analysis [56-58]: the Bayesian information criterion (BIC), the Akaike information criterion (AIC), entropy, and group composition. AIC relies on information theory to assess the relative information value of the model by considering the maximum likelihood estimate and the number of parameters within the model [59]. Similar to AIC, BIC originates from the Bayesian framework and can be interpreted as the posterior probability of a model based on the observed data [60]. Both statistics aim to identify the most informative model by balancing between goodness-of-fit and model complexity. However, BIC imposes a stronger penalty for model complexity compared with AIC, taking into account the sample size [61]. The goodness-of-fit and penalty terms are summed to compute AIC and BIC values, where smaller values indicate better-fitting models [62-64]. Additionally, entropy assesses the classification accuracy of the model by summarizing the likelihood of each participant being correctly classified [57]. With values ranging from 0 to 1, higher entropy values indicate more precise classification, typically considered satisfactory when exceeding 0.8 [65]. We also analyzed the group composition (ie, the percentage of the population represented in each subgroup), ensuring that each subgroup represented at least 5% of the total sample [58]. Second, we determined the shapes of each trajectory by specifying their functional forms (eg, linear and cubic). Starting with a cubic specification (up to 3 degrees), we iteratively dropped nonsignificant (*P*>.05) polynomial terms until only significant ones remained [56]. In the count model part, linear terms were retained regardless of their statistical significance. Third, after identifying the optimal number of trajectories and their shapes, we used the average posterior probabilities (APPs) of group membership to validate the selected model. The APP measures the average probability of each participant belonging to their assigned group and should ideally be at least 0.7 for each group to ensure robustness [58].

After identifying the best-fitting model, we assigned users to their respective trajectory groups. Subsequently, summary descriptive statistics of linguistic features during each period were computed and graphed for each user group. Specifically, we compared linguistic frequencies between groups across different periods. As a result of the skewed and excessively zero-inflated distribution of LIWC frequency, parametric tests such as *t* tests or ANOVA may not be appropriate, as they violate their assumptions and can reduce the robustness of nonparametric tests such as the median or Wilcoxon-Mann-Whitney test. Therefore, Poisson regression modeling was recommended for its improved interpretability of data and comparability among potential models [66]. In the Poisson regression model, group membership was included as an independent variable, while the frequency of each linguistic feature in each period served as the dependent variable. The results of the Poisson model provided rate ratios (RRs) along with SEs, indicating the relative changes in counts of the outcomes between the groups.

In the final analysis, our goal was to identify linguistic markers that could differentiate between groups of users exhibiting different trajectories. In the context of suicidal text analysis, linguistic markers, or linguistic distinguishers, are language features (eg, LIWC categories) extracted from texts that substantially distinguish users or posts with varying risk statuses [17,49]. To identify these features, we analyzed the linguistic profiles of high- and low-risk users and examined which words could indicate their respective suicide risk levels. As the markers were not intended to predict users’ group membership, a temporal sequence (eg, baseline linguistic data) was not necessary. We utilized data from the 7 periods and followed these steps to identify potential linguistic markers. First, we identified linguistic features from the periods that exhibited significant (*P*≤.05) between-group differences based on the results of Poisson regression, considering them as potential markers. Second, to prevent duplication, we excluded high-level LIWC categories that have hierarchical relationships with each other (eg, ppron includes I, we, you, she/he, they). Third, we used Lasso logistic regression with cross-validation to determine the optimal penalty parameter, aiming to mitigate collinearity among the remaining linguistic measures [67]. Given the lack of a theoretical basis for estimating post-selection coefficients with nonlinear Lasso models [67], we utilized the Lasso model solely for model selection purposes to filter out redundant variables. The remaining variables were then integrated into the best-fitting GBTM, and their associations with group membership were assessed using multivariate logistic regression [68]. In this analysis, we computed odds ratios (ORs), where potential linguistic features served as independent variables and group memberships as the dependent variable. Significance levels were determined at a *P* value ≤.05. Data extraction was performed using Python (Python Foundation), while data analysis was conducted using Stata/SE 16.1 (StataCorp LLC) along with the Traj plugin for trajectory analysis.

### Ethical Considerations

The data used in this study were obtained from publicly accessible posts on the r/SuicideWatch subreddit through purely observational and nonintrusive means. The raw data did not contain personally identifiable information. To uphold user privacy and confidentiality, selected posts were deidentified before analysis. This involved removing any identifying information such as names, genders, ages, addresses, and links from the post content. We maintained annotated user data separately from the raw data and stored them on secure servers, linked only through anonymous IDs. Furthermore, all examples presented in Multimedia Appendix 1 were anonymized and paraphrased to safeguard user privacy, following the framework outlined by Bruckman [69]. As publicly available data were utilized, this study fell outside the purview of ethical review by the City University of Hong Kong Research Committee, for which an exemption was obtained.

## Results

### Post Trajectories on the r/SuicideWatch Subreddit Throughout the COVID-19 Pandemic

To determine post trajectories, we evaluated the model fit statistics for 2- to 5-group solutions of the GBTM to identify the optimal number of trajectory groups (Table 1). As the number of groups increased from 2, we noted that both the AIC and BIC values tended to increase, while entropy decreased. Additionally, starting from the 3-group model, some group compositions did not meet the 5% threshold. Therefore, we selected the 2-group model as the optimal choice, with AIC and BIC values of –39,605.31 and –39,659.12, respectively, and an entropy of 0.96. Further analysis indicated that the 2-group solution, using cubic and quadratic functions in the count model and 2 cubic functions in the inflation model, resulted in all polynomial terms being statistically significant (Table 2). The APPs for groups 1 and 2 were 0.95 and 0.99, respectively, indicating strong alignment between users and their assigned groups within this 2-group ZIP model.

**Table 1:** Fit statistics for the 2- to 5-group solution–based trajectory modeling of post volume among r/SuicideWatch users throughout the COVID-19 pandemic.

Model	AIC^a^	BIC^b^	Entropy^c^	Composition^d^, n/N (%)
2-Group model	–39,605.31	–39,659.12	0.96	744/6163 (12.07)/5419/6163 (87.93)
3-Group model	–36,862.86	–36,940.22	0.892	1562/6163 (25.34)/4379/6163 (71.05)/222/6163 (3.60)
4-Group model	–35,890.32	–35,981.12	0.836	625/6163 (10.14)/1502/6163 (24.37)/3936/6163 (63.87)/100/6163 (1.62)
5-Group model	–36,099.67	–36,190.48	0.873	293/6163 (4.75)/1699/6163 (27.57)/91/6163 (1.48)/4003/6163 (64.95)/77/6163 (1.25)

^a^AIC: Akaike information criterion (a lower value is better).

^b^BIC: Bayesian information criterion (a lower value is better).

^c^Entropy (a value >0.8 is better).

^d^Group composition (the percentage of the population represented in each subgroup should exceed 5%).

**Table 2:** Parameter estimates for the 2-group zero-inflated group–based trajectory modeling of post volume among r/SuicideWatch users throughout the COVID-19 pandemic.

Group and parameter			Estimate	SE		*t* value (*df*)		*P* value
**1 (count)**								
	Intercept	2.62		0.01	214.7 (743)		<.001	
	Linear	0.1		0.03	3.63 (743)		<.001	
	Quadratic	–0.44		0.02	–24.52 (743)		<.001	
	Cubic	–0.22		0.02	–10.56 (743)		<.001	
**2 (count)**								
	Intercept	0.59		0.01	42.46 (5418)		<.001	
	Linear	–0.14		0.02	–5.99 (5418)		<.001	
	Quadratic	–0.12		0.02	–5.07 (5418)		<.001	
**1 (inflation)**								
	Alpha0	–0.19		0.05	–3.78 (743)		<.001	
	Alpha1	–1.85		0.1	–18.4 (743)		<.001	
	Alpha2	1.49		0.06	24.17 (743)		<.001	
	Alpha3	0.75		0.07	10.8 (743)		<.001	
**2 (inflation)**								
	Alpha0	–0.34		0.03	–13.16 (5418)		<.001	
	Alpha1	–2.31		0.06	–37.54 (5418)		<.001	
	Alpha2	2.63		0.04	58.83 (5418)		<.001	
	Alpha3	1.49		0.05	27.77 (5418)		<.001	

**Figure 1:**
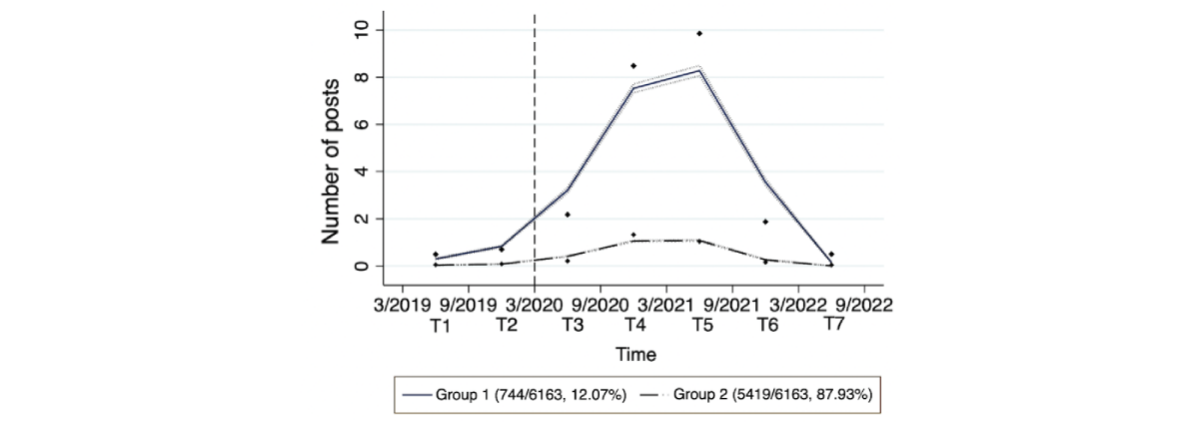
Trajectories of post volume among r/SuicideWatch users throughout the COVID-19 pandemic.

Figure 1 depicts the post volume trajectories across the COVID-19 pandemic for the 2 identified groups of users. Group 1, designated as the “high risk of suicide” group, consisted of 744 (12.07%) users. Their post volume on r/SuicideWatch showed a gradual increase during the 2 prepandemic periods, followed by a rapid acceleration after the pandemic began. This trend peaked approximately 1 year after the pandemic outbreak and subsequently declined, returning to its initial level during the second year of the pandemic. Group 2, identified as the “low risk of suicide” group, comprised the majority of users (5419/6163, 87.93%). This group exhibited a slight increase in post volume on r/SuicideWatch following the pandemic outbreak, followed by stabilization and eventual recovery. Throughout the pandemic, group 2 maintained a relatively low post volume on the subreddit.

### Linguistic Feature Analysis

The summary distribution of frequency of use in LIWC for the 2 groups can be found in Multimedia Appendix 2. Similar to the distribution of post volume, we observed a zero-inflated phenomenon for these linguistic features across periods. Therefore, descriptive statistics including the median, first quartile, and third quartile were used. By plotting the median frequency trend for each included LIWC feature throughout the pandemic for the 2 groups, trends by category were illustrated (Multimedia Appendix 3). We observed the following: (1) During the year before the pandemic (T1: March 2019-September 2019 and T2: September 2019-March 2020), word frequency was generally low for both groups. (2) Throughout the pandemic (T3-T6: March 2020-March 2022), words related to cognitive processes, perceptual processes, biological processes, and personal concerns showed relatively lower frequency compared with personal pronouns, affective processes, social processes, drives, relativity, and time orientations. (3) During the first year of the pandemic (T3-T4: March 2020-March 2021), both groups exhibited sharp increases in word frequency. (4) During the second year of the pandemic (T5-T6: March 2021-March 2022), the high-risk group continued to experience a slower increase until reaching a peak and subsequent decrease, while the low-risk group’s frequency decreased. (5) Moving into the third year of the pandemic (T7: March 2022-September 2022), word frequency returned to prepandemic levels in both groups. Despite both groups showing increased use of most word types during the pandemic, the high-risk group exhibited a longer-lasting increase with a peak lagging behind that of the low-risk group. This suggests that the pandemic had a more enduring impact on high-risk users.

**Figure 2:**
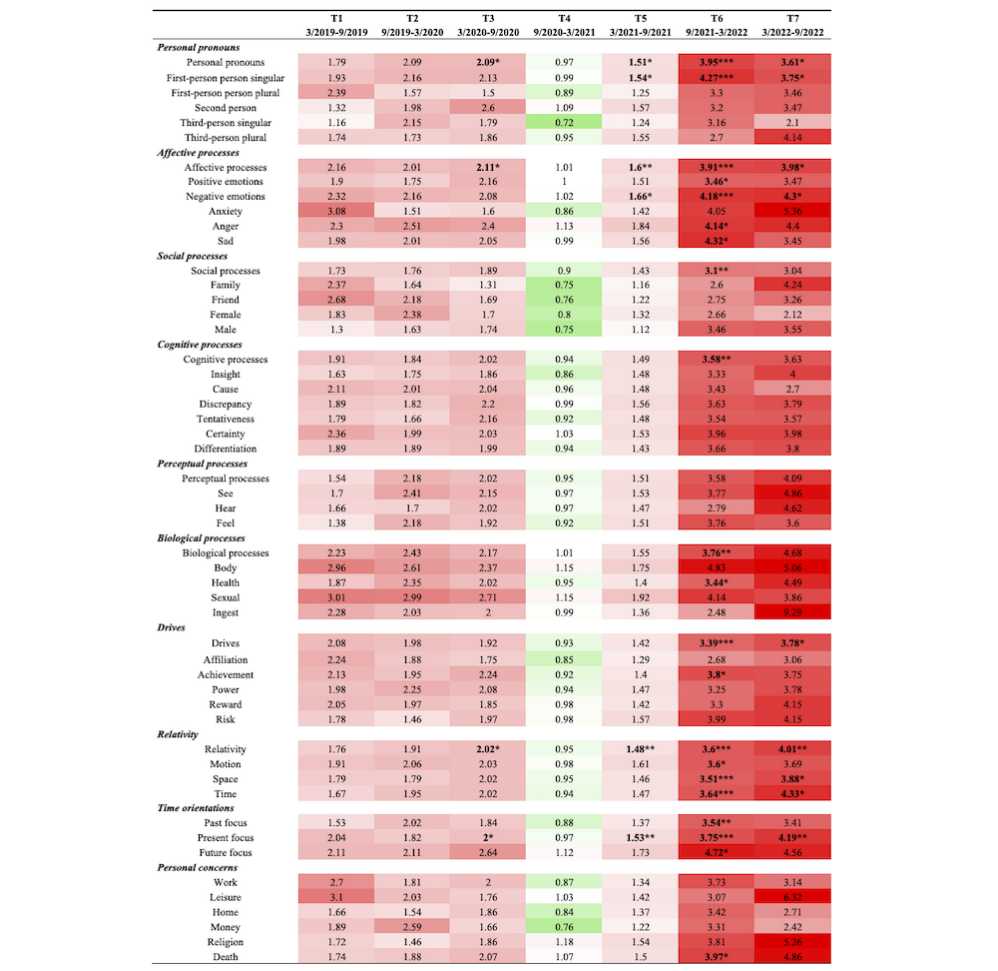
Visualization of Poisson regression results comparing LIWC frequency between r/SuicideWatch users in the high- and low-risk groups throughout the COVID-19 pandemic (low-risk group as a reference; red: higher frequency in the high-risk group; green: lower frequency in the high-risk group). LIWC: Linguistic Inquiry and Word Count. *P<.05; **P<.01; ***P<.001.

The results of between-group comparisons using Poisson regression (with the low-risk group as the reference) are depicted in Figure 2. In general, the high-risk group utilized most types of words more frequently than the low-risk group both before and during the initial 6 months of the pandemic (illustrated in red for T1-T3). Later, in the second half of the pandemic, their differences narrowed and even reversed (as shown in green during T4), with both groups demonstrating increased word use. Subsequently, the high-risk group once again surpassed the low-risk group, and these differences grew larger in the subsequent periods (as indicated in deeper red from T5 to T7). This pattern corresponded with the plotted trend, where the high-risk group exhibited a prolonged increase and a delayed peak following the rise during T4, whereas the frequency of the low-risk group quickly decreased and returned to its initial level.

Statistical differences in the frequency of word use were primarily observed after the pandemic outbreak (Figure 2). During T3 (March 2020-September 2020), the high-risk group showed significantly more frequent use of words related to personal pronouns (RR 2.09, SE 0.73, *P*=.03), affective processes (RR 2.11, SE 0.73, *P*=.03), relativity (RR 2.02, SE 0.55, *P*=.01), and present focus (RR 2.00, SE 0.54, *P*=.01) compared with the low-risk group. During T5 (March 2021-September 2021), posts in the high-risk group also exhibited higher frequencies of words related to personal pronouns (RR 1.51, SE 0.26, *P*=.02), first-person singular (RR 1.54, SE 0.31, *P*=.03), affective processes (RR 1.60, SE 0.27, *P*=.005), negative emotions (RR 1.66, SE 0.35, *P*=.02), relativity (RR 1.48, SE 0.20, *P*=.005), and present focus (RR 1.53, SE 0.20, *P*=.001). During T6 (September 2021-March 2022), 21 types of words across categories such as personal pronouns; affective, social, cognitive, and biological processes; drives; relativity; time orientations; and personal concerns showed higher frequencies in the high-risk group compared with the low-risk group. Meanwhile, in T7 (March 2022-September 2022), the high-risk group exhibited higher frequencies of words related to personal pronouns (RR 3.61, SE 2.08, *P*=.03), first-person singular (RR 3.75, SE 2.38, *P*=.04), affective processes (RR 3.98, SE 2.22, *P*=.01), negative emotions (RR 4.3, SE 3.02, *P*=.04), drives (RR 3.78, SE 2.35, *P*=.03), relativity (RR 4.01, SE 1.75, *P*=.001), space (RR 3.88, SE 2.66, *P*=.04), time (RR 4.33, SE 2.76, *P*=.02), and present focus (RR 4.19, SE 1.8, *P*=.001).

To investigate the linguistic markers that could distinguish group membership, we identified 21 word types that significantly (*P*≤.05) differed between the 2 groups during the last 3 periods (T5, T6, and T7). These word types include personal pronouns, first-person singular, affective processes, positive emotions, negative emotions, anger, sadness, social processes, cognitive processes, biological processes, health, drives, achievement, relativity, motion, space, time, past focus, present focus, future focus, and death. The word types with the most observed differences were selected as potential linguistic markers for further examination. Then, we omitted 6 word types (ie, personal pronouns, affective processes, negative emotions, biological processes, drives, and relativity) due to their hierarchical relationship with their subcategory words to avoid duplication. To better fit the multivariate logistic regression, we calculated a binary measure for each of the remaining 15 potential markers, indicating no use (0) or use (1) of the word. We calculated the average frequency of each word across T5, T6, and T7, and then dichotomized these averages. Averaged values of 0 were retained as 0, indicating no use of the word during T5, T6, and T7. For averaged values greater than 0, we recoded the value as 1, indicating that the word was used at least once during T5, T6, and T7, regardless of the actual frequency. To mitigate collinearity among the 15 words, we used lasso regression for variable selection. Ultimately, we omitted 3 word types—specifically, the first-person singular, space, and time—leaving us with 12 linguistic features: positive emotions, anger, sadness, social processes, cognitive processes, health, achievement, motion, past focus, present focus, future focus, and death.

Table 3 presents the results of the multivariate logistic regression, incorporating potential linguistic markers into the 2-group GBTM. This analysis models the odds of being in the high-risk group based on the usage of potential linguistic features, with no use of the word serving as the reference. The final model indicated that 9 linguistic features emerged as significant (*P*≤.05) markers distinguishing the 2 groups. Notably, using words related to cognitive processes and present focus during the later COVID-19 periods had lower odds of being in the high-risk group compared with not using these words (OR_cognitive processes_ 0.06, SE 0.85, *P*<.001; OR_present focus_ 0.03, SE 0.85, *P*<.001). This indicates that the use of these words was associated with being in the low-risk group. Contrastingly, the odds of being in the high-risk group were substantially higher when using words related to anger, sadness, health, achievement, motion, future focus, and death, compared with not using these words (OR_anger_ 3.23, SE 0.29, *P*<.001; OR_sadness_ 3.23, SE 0.25, *P*<.001; OR_health_ 2.56, SE 0.33, *P*=.005; OR_achievement_ 1.67, SE 0.26, *P*=.049; OR_motion_ 4.17, SE 0.37, *P*<.001; OR_future focus_ 2.86, SE 0.3, *P*<.001; OR_death_ 4.35, SE 0.26, *P*<.001). The results illustrated that these 7 words, used 1 year after the pandemic outbreak, were linguistic markers for being in the high-risk group. Multimedia Appendix 1 provides examples of posts that high-risk users published 1 year after the pandemic outbreak.

**Table 3:** Odds ratios from multivariate logistic regression to identify linguistic markers that discriminate r/SuicideWatch users in the high- and low-risk groups.

Linguistic markers	Odds ratio	SE	*t* value (*df*)	*P* value
Constant	14.83	0.08	32.37 (6162)	<.001
Positive emotions	1.79	0.44	1.34 (6162)	.18
Anger	3.23	0.29	4.12 (6162)	<.001
Sad	3.23	0.25	4.75 (6162)	<.001
Social processes	0.56	0.53	–1.11 (6162)	.27
Cognitive processes	0.06	0.85	–3.38 (6162)	<.001
Health	2.56	0.33	2.84 (6162)	.005
Achievement	1.67	0.26	1.97 (6162)	.049
Motion	4.17	0.37	3.81 (6162)	<.001
Past focus	1.82	0.40	1.48 (6162)	.14
Present focus	0.03	0.85	–4.27 (6162)	<.001
Future focus	2.86	0.3	3.52 (6162)	<.001
Death	4.35	0.26	5.61 (6162)	<.001

## Discussion

### Principal Findings

To the best of our knowledge, this work is the first to address heterogeneity in suicide risk among social media users by incorporating the temporal characteristics of suicide. Based on the 2 identified trajectories of post volume throughout the COVID-19 pandemic, users on the r/SuicideWatch subreddit were divided into the “high risk of suicide” group (744/6163, 12.07%), characterized by a sharp and lasting increase in post volume, and the “low risk of suicide” group (5419/6163, 87.93%), characterized by a consistently low and mild increase in post volume during the pandemic. In terms of linguistic features, the 2 groups exhibited distinct frequency trends throughout the pandemic. The high-risk group demonstrated longer-lasting increases and lagged peaks in most linguistic frequencies. Contrarily, the low-risk group displayed different trends. Notably, the use of words related to anger, sadness, health, achievement, motion, future focus, and death 1 year after the pandemic outbreak emerged as markers for membership in the high-risk group. Conversely, words associated with cognitive processes and present focus were identified as linguistic markers for the low-risk group.

Across the pre- and peripandemic periods, this study identified 2 distinct patterns of change in suicide risk among r/SuicideWatch users based on trajectory modeling of their post volume. These findings underscore the heterogeneity in suicide risk among r/SuicideWatch users from a longitudinal perspective during the pandemic. Users’ participation in subreddits, including posting frequency, commenting habits, and emotional expression, was influenced by significant pandemic events [38], particularly its progression in Western countries such as the US, the UK, Canada, Australia, and Germany, where a majority of Redditors originate [70]. Both groups of users exhibited immediate increases in post volume following the onset of the COVID-19 pandemic in March 2020. However, post volume returned to prepandemic levels in later stages, around September 2021, as many Western countries began to resume normalcy [71]. According to the fluid vulnerability theory [40], an environmental stressor can trigger a suicidal response within individuals who have predispositions to such reactions. While the half-year intervals may not fully capture users’ detailed responses to the pandemic or fluctuations in their suicidal episodes, the heightened posting activity observed in both groups following the pandemic’s onset suggests an overall increase in their suicide risk. Therefore, the ongoing pandemic and its repercussions may serve as a persistent environmental stressor for users. Importantly, the high-risk group exhibited significantly greater increases in post volume during the pandemic (T3-T5: March 2020-September 2021) compared with the low-risk group. This suggests that the onset of suicidal episodes was more pronounced among the high-risk group than the low-risk group. The finding of users’ heterogeneity in suicide risk can be explained by the interaction between one’s baseline and acute risk of suicide, as proposed by the fluid vulnerability theory [41]. Individuals in the high-risk group may have a higher baseline risk due to underlying vulnerabilities, making their suicidal tendencies more readily activated compared with those in the low-risk group, who have fewer vulnerabilities and a lower baseline risk. The higher level of predispositions among high-risk users also renders them more vulnerable to the adverse impacts of the pandemic. This vulnerability activates heightened risks in various domains including cognition (eg, hopelessness), emotion (eg, depression), behavior (eg, social withdrawal), and physiology (eg, sleep disturbances), contributing to their increased acute risk. The higher baseline and acute risks motivate high-risk users to express their heightened concerns, seek support, and exchange information online, leading to a significant increase in social media engagement [72]. By contrast, the low-risk group, which showed consistently low and mild increases in post volume, likely represents the majority less predisposed to suicide risk, indicating greater resilience to the pandemic. Therefore, they may perceive the pandemic as less threatening and experience fewer burdens related to cognitive, emotional, behavioral, and physiological factors. With fewer concerns to share, they exhibited only a mild and minimal increase in post volume. Our findings underscore the heterogeneity in patterns of suicide risk change during the pandemic within this population, highlighting the importance of considering users’ individual differences and the temporal dynamics of suicide in future studies using social media data.

Additionally, this study observed differences in the trends of linguistic features between the high- and low-risk groups. During the first year of the pandemic (T3-T4: March 2020-March 2021), both groups significantly increased their use of words related to personal pronouns, positive and negative emotions, social processes, drives, relativity, and time orientations compared with other word categories, indicating broader topics of interest during this period [73]. However, the increase in linguistic frequency continued at a slower pace in the high-risk group before reaching a peak and returning to its original volume (T5-T7: March 2021-September 2022), whereas the low-risk group experienced an early, mild peak followed by an immediate decrease. This divergent trend highlights that most statistical differences in linguistic frequency between the 2 groups became evident 1 year after the outbreak of the pandemic (T5-T7: March 2021-September 2022), indicating that the impact of the pandemic on the high-risk group was more prolonged and delayed compared with the low-risk group. This finding not only underscores the heterogeneity between the 2 groups but also highlights that high-risk users have experienced prolonged stress and heightened sensitivity during the pandemic.

To better identify users at high risk of suicide and understand their underlying concerns, we examined linguistic markers based on several features that showed between-group differences 1 year into the pandemic. Specifically, words related to anger, sadness, health, achievement, motion, future focus, and death were identified as linguistic markers for the high-risk group, which partially aligns with previous findings [36,49]. We delved deeper into the post content of high-risk users to grasp the context in which these linguistic markers were used. Words related to anger and sadness were used by high-risk users to express agitation and hopelessness concerning the overwhelming impact of the pandemic, emotions strongly linked with an increased risk of suicidal thoughts and behaviors [74-76]. When discussing health and motion, high-risk users conveyed heightened concerns about their physical well-being and limitations in movement due to pandemic-related lockdowns [37]. Additionally, they used achievement-related words to express feelings of failure in meeting their goals and fulfilling their need for social recognition. These users may place high demands on themselves, striving to accomplish difficult tasks and meet high standards, which can increase their vulnerability to depression and suicidal behaviors [17,77]. The widespread economic losses, unemployment, and disruptions in educational settings caused by the pandemic further impeded their ability to achieve success, leading to lowered self-esteem, depressive mood, and heightened suicidal risk [78]. Additionally, we discovered that words related to future focus served as linguistic markers for the high-risk group. While previous studies have noted that suicidal individuals often emphasize present-focused words, reflecting their hopelessness about the future and acute concerns about their current state [49,79], this pattern may differ during the pandemic. High-risk users articulated their apprehensions about an uncertain and uncontrollable future amid the evolving pandemic, as exemplified in the texts (Multimedia Appendix 1). Additionally, the high-risk group used more words related to death. In addition to referencing suicide or hopelessness, this marker also indicated their perceived threats from virus infections, death cases, or the loss of loved ones during the pandemic [37,80].

Our findings have significant implications for managing suicide issues during future public health crises. By analyzing social media posts, we identified a small percentage of users at high risk of suicide who appear particularly sensitive and vulnerable to pandemic-related events or similar public health crises in the future. Although the majority are at low risk of suicide, these results underscore serious concerns, as high-risk users may be poised to progress to the next stage of suicidal ideation or take action [36]. Therefore, it is crucial to pay particular attention to this subset of users to alleviate their difficulties in such situations. Moreover, the active posting and disclosure by these high-risk users may lead to “suicidal contagion” affecting low-risk users, potentially propagating suicidal tendencies within online communities [81]. Therefore, ongoing surveillance, screening, and timely intervention during public health crises are necessary to prevent this issue. Furthermore, the distinct linguistic patterns observed in the 2 groups in this study can serve as a foundation for understanding the underlying concerns contributing to these users’ suicide risk, thereby aiding in the development of targeted interventions. The identified language markers for the high-risk group can also serve as a basis for screening high-risk individuals in future pandemic-like events.

Additionally, this study has several limitations. First, aside from users disclosing their own suicidal issues, r/SuicideWatch includes posts about others’ suicide risk, providing assistance to those in distress, and disseminating research messages [24]. Although the percentage of these posts was small in our manual screening of selected posts (18/3372, 0.53%, sampled posts), future studies are advised to mitigate this noise or incorporate users’ other online behaviors (eg, commenting frequency and post length) to more accurately assess users’ suicide risk. Moreover, a significant portion of users in our data set transitioned from other subreddits to r/SuicideWatch following the onset of the pandemic, starting with 0 post volume in periods preceding their initial posts (eg, 2 prepandemic periods). Future studies could track users’ earlier psychosocial characteristics on other subreddits to identify indicators that might foreshadow their shift toward actively discussing suicidal concerns on r/SuicideWatch. Second, we utilized seven 6-month intervals as the time frames for capturing post volume and linguistic frequency, which may have been too lengthy to capture specific fluctuations. Nan et al [82] also utilized 6-month intervals and identified a 2-trajectory model for changes in suicidal ideation throughout the pandemic using scores from multiple-item scales as the trajectory variable. However, using shorter intervals (eg, 2-6 weeks) can reveal more trajectories, as it considers minor but significant differences rather than averaging them in the analysis [83,84]. Given the frequent release of pandemic-related news and information (eg, daily reports), users shared real-time reactions to these updates in their posts, potentially reflecting immediate changes in their suicidal thoughts or behaviors, a nuance that might not have been fully captured in our study [38]. Future studies could benefit from shorter time intervals to capture more nuanced and continuous changes in suicide risk, potentially revealing diverse trajectories of suicidal ideation. Third, due to the anonymity of Reddit data, our access was restricted to users’ demographics (eg, country or region, age, and sex). Consequently, these factors could not be included as potential covariates for modeling trajectory groups or for comparing the demographic compositions between high- and low-risk user groups. We also acknowledge the potential confounding impact of varying pandemic waves and government control policies across different countries, which we were unable to explore due to the lack of geographical information from users. Future studies should aim to investigate these factors while maintaining the integrity of data characterized by high self-disclosure and authenticity [14]. Additionally, our analysis focused exclusively on Reddit data from a Western context [70]. Cross-cultural validation using data from other platforms, such as Weibo, will be crucial to enhance the generalizability of findings and consider cultural and national policy influences.

### Conclusions

This study used social media posts to demonstrate the heterogeneous patterns of change in suicide risk during the COVID-19 pandemic. A group of Reddit users at high risk of suicide was identified, characterized by a sharp and sustained increase in post volume. These high-risk users exhibited distinct linguistic patterns, particularly in their use of words related to anger, sadness, health, achievement, motion, future focus, and death during the later stages of the pandemic. Our findings underscore the importance of recognizing users’ heterogeneity in long-term suicide risk. Real-time surveillance of suicide risk using social media data during future public health crises is essential to provide timely support to individuals potentially at high risk of suicide.

## Appendix

Multimedia Appendix 1 Examples of linguistic markers for users in the high-risk group.

Multimedia Appendix 2 Summary distribution of LIWC frequency for r/SuicideWatch users in the high- and low-risk groups during each period (median [Q1, Q3]). LIWC: Linguistic Inquiry and Word Count.

Multimedia Appendix 3 Trends of LIWC frequency by category for r/SuicideWatch users in the high- and low-risk groups throughout the COVID-19 pandemic (based on the median). LIWC: Linguistic Inquiry and Word Count.

## Abbreviations

AIC: Akaike information criterion
APP: average posterior probability
BIC: Bayesian information criterion
CNORM: censored normal
GBTM: group-based trajectory modeling
LIWC: Linguistic Inquiry and Word Count
OR: odds ratio
ZIP: zero-inflated Poisson

## Data Availability

The data set used in this study is available from the corresponding author upon request.
