# Supplementary material for: Identifying Reddit Users at a High Risk of Suicide and Their Linguistic Features During the COVID-19 Pandemic: Growth-Based Trajectory Model
Source: J Med Internet Res. 2024 Aug 8;26:e48907. doi: 10.2196/48907 (PMC11342008; doi:10.2196/48907)
Supplement: Multimedia Appendix 1 [file jmir_v26i1e48907_app1.docx]

Multimedia Appendix 3. Examples of linguistic markers for users in the high-risk group.

| Linguistic markers | Keywords | Textual example (title: content)^a^ |
| --- | --- | --- |
| - Anger | - hate, shit | *Covid has been hard on all of us:...Im [I am] so tired of normal people telling me how hard covid is for all of them and how hard it was to not socialise and see their friends. Ive [I have] been* ***alone*** *all of my life Ive [I have]* ***never hung out with anyone*** *outside of school. I don't have a social life. I don’t like being in public but I* ***hate*** *being alone. Im [I am] so tired of these people saying how hard it was to not talk to their friends in 4 months. I know I sound like I bitter jealous piece of* ***shit*** *because I am but Im [I am] tired of normal people I really am. At least now they know how it feels to be unattractive and mentally ill* ***alone*** *and* ***sad*** *for months at a time.* |
| - Sadness | - alone, sad |  |
| - Health | - pain, health, throat, fever, cough | *I hope I die: Just tested positive for covid and* ***am having chest pains****. I am fantasizing a quick death, but am worried if it's the slow can't breathe kind...I have a pending biopsy situation for another* ***health issue*** *and spent my birthday two days ago* ***sick*** *and in isolation...They want to live and I don't...*  *I'm getting my covid test results tomorrow:...I've been feeling like absolute shit for the past few weeks. I've had* ***sore throat,*** *a possible* ***fever****, and now I have* ***cough****...if I'm positive, I'll kill myself. I used to be afraid of death...but not anymore. I'll use any method I can as long as I don't see another day...* |
| - Achievement | - succeeding, achieve | *I just don't want to wake up anymore: I am tired of waking up every day still not knowing what I want to do with my life...I am tired of waking up and feel like shit. I am tired of worrying about making mistakes with the things I say, do, don't say or don't do...I do have my family, friends and a girlfriend but even with all of that I still don't want to be here...I feel like I let all of them down by not* ***succeeding****. Everyone says get a job or change things like it's that easy. Life is just tough and I am tired of feeling like I am going up against so much before I can* ***achieve*** *what I want. Especially now with Covid things are even more miserable than before.* |
| - Motion | - travel, quarantine | *I wish people did stop caring about me so I could* ***leave****:...Everyday I hope things get better, but then you see countries like Canada go into* ***lockdown****...Sure I still worked, but I had a huge passion for* ***traveling****. It was something I would always look forward to doing. Now I can't do that because of* ***travel bans or restrictions****. I can't afford to* ***travel*** *and then be told to* ***quarantine*** *for two weeks...I am sorry I can't see the so called “light at the end of the tunnel.”* |
| - Future focus | - going, will | *Everyone cares for each other but it's selfish to care about yourself:...Masking, distancing, all of that stuff you guys know. Lately I have had bad thoughts that what we are going through is* ***never going to end****. That we* ***will*** *always have* *to live with these restrictions...Everyday I keep waking up to covid this and covid that and I am about at the end of my rope...*  *I just don't want to wake up anymore:...I am tired of waking up worrying about what's* ***going*** *to happen today...* |
| - Death | - die, suicide | *Why is it so fucking hard to die?*: *I was happy that I got covid and can* ***die*** *without doing anything...I got the “good” news which isn’t the delta virus. I just want to* ***die****. I fucking hate being alive...*  *800,000 people* ***die*** *from* ***suicide*** *worldwide every year and only 72,000 people have* ***died*** *from covid since it first appeared.* |
