# Supplementary material for: Identifying Reddit Users at a High Risk of Suicide and Their Linguistic Features During the COVID-19 Pandemic: Growth-Based Trajectory Model
Source: J Med Internet Res. 2024 Aug 8;26:e48907. doi: 10.2196/48907 (PMC11342008; doi:10.2196/48907)
Supplement: Multimedia Appendix 2 [file jmir_v26i1e48907_app2.docx]

**Multimedia Appendix 1**. Summary distribution of LIWC frequency for r/SuicideWatch users in the high- and low-risk groups during each period (median [Q1, Q3]).

|  | T1  (3/2019-9/2019) | | T2  (9/2019-3/2020) | | T3  (3/2020-9/2020) | | T4  (9/2020-3/2021) | | T5  (3/2021-9/2021) | | T6  (9/2021-3/2022) | | T7  (3/2022-9/2022) | |
| --- | --- | --- | --- | --- | --- | --- | --- | --- | --- | --- | --- | --- | --- | --- |
|  | G1 | G2 | G1 | G2 | G1 | G2 | G1 | G2 | G1 | G2 | G1 | G2 | G1 | G2 |
| ***Personal pronouns*** | | | | | | | | | | | | | | |
| Personal pronouns | 0(0,0) | 0(0,0) | 0(0,0) | 0(0,0) | 0(0,0) | 0(0,0) | 0.046(0, 0.067) | 0.039(0, 0.073) | 0.055(0, 0.076) | 0(0,0.063) | 0(0,0.042) | 0(0,0) | 0(0,0) | 0(0,0) |
| First-person singular | 0(0,0) | 0(0,0) | 0(0,0) | 0(0,0) | 0(0,0) | 0(0,0) | 0.029(0, 0.046) | 0.026(0, 0.049) | 0.039(0, 0.053) | 0(0,0.043) | 0(0,0.031) | 0(0,0) | 0(0,0) | 0(0,0) |
| First-person plural | 0(0,0) | 0(0,0) | 0(0,0) | 0(0,0) | 0(0,0) | 0(0,0) | 0(0,0.001) | 0(0,0) | 0(0,0.001) | 0(0,0) | 0(0,0) | 0(0,0) | 0(0,0) | 0(0,0) |
| Second person | 0(0,0) | 0(0,0) | 0(0,0) | 0(0,0) | 0(0,0) | 0(0,0) | 0(0,0.006) | 0(0,0.002) | 0.002(0, 0.006) | 0(0,0) | 0(0,0) | 0(0,0) | 0(0,0) | 0(0,0) |
| Third-person singular | 0(0,0) | 0(0,0) | 0(0,0) | 0(0,0) | 0(0,0) | 0(0,0) | 0(0,0.003) | 0(0,0) | 0(0,0.005) | 0(0,0) | 0(0,0) | 0(0,0) | 0(0,0) | 0(0,0) |
| Third-person plural | 0(0,0) | 0(0,0) | 0(0,0) | 0(0,0) | 0(0,0) | 0(0,0) | 0(0,0.001) | 0(0,0.005) | 0.003(0, 0.007) | 0(0,0.002) | 0(0,0) | 0(0,0) | 0(0,0) | 0(0,0) |
| ***Affective processes*** | | | | | | | | | | | | | | |
| Affective processes | 0(0,0) | 0(0,0) | 0(0,0) | 0(0,0) | 0(0,0) | 0(0,0) | 0.058(0, 0.076) | 0.049(0, 0.074) | 0.067(0, 0.082) | 0(0,0.068) | 0(0,0.057) | 0(0,0) | 0(0,0) | 0(0,0) |
| Positive emotions | 0(0,0) | 0(0,0) | 0(0,0) | 0(0,0) | 0(0,0) | 0(0,0) | 0.017(0, 0.027) | 0.012(0, 0.027) | 0.021(0, 0.029) | 0(0,0.023) | 0(0,0.014) | 0(0,0) | 0(0,0) | 0(0,0) |
| Negative emotions | 0(0,0) | 0(0,0) | 0(0,0) | 0(0,0) | 0(0,0) | 0(0,0) | 0.033(0, 0.048) | 0.025(0, 0.046) | 0.041(0, 0.054) | 0(0,0.042) | 0(0,0.033) | 0(0,0) | 0(0,0) | 0(0,0) |
| Anxiety | 0(0,0) | 0(0,0) | 0(0,0) | 0(0,0) | 0(0,0) | 0(0,0) | 0.002(0, 0.005) | 0(0,0.005) | 0.003(0, 0.007) | 0(0,0.004) | 0(0,0) | 0(0,0) | 0(0,0) | 0(0,0) |
| Anger | 0(0,0) | 0(0,0) | 0(0,0) | 0(0,0) | 0(0,0) | 0(0,0) | 0.008(0, 0.018) | 0(0,0.015) | 0.012(0, 0.021) | 0(0,0.011) | 0(0,0.008) | 0(0,0) | 0(0,0) | 0(0,0) |
| Sad | 0(0,0) | 0(0,0) | 0(0,0) | 0(0,0) | 0(0,0) | 0(0,0) | 0.007(0, 0.013) | 0(0,0.013) | 0.009(0, 0.015) | 0(0,0.01) | 0(0,0.006) | 0(0,0) | 0(0,0) | 0(0,0) |
| ***Social processes*** | | | | | | | | | | | | | | |
| Social processes | 0(0,0) | 0(0,0) | 0(0,0) | 0(0,0) | 0(0,0) | 0(0,0) | 0.037(0, 0.063) | 0.032(0, 0.068) | 0.05(0, 0.069) | 0(0,0.058) | 0(0,0.029) | 0(0,0) | 0(0,0) | 0(0,0) |
| Family | 0(0,0) | 0(0,0) | 0(0,0) | 0(0,0) | 0(0,0) | 0(0,0) | 0(0,0.004) | 0(0,0.004) | 0.001(0, 0.005) | 0(0,0) | 0(0,0) | 0(0,0) | 0(0,0) | 0(0,0) |
| Friend | 0(0,0) | 0(0,0) | 0(0,0) | 0(0,0) | 0(0,0) | 0(0,0) | 0(0,0. 003) | 0(0,0.003) | 0.001(0, 0.004) | 0(0,0) | 0(0,0) | 0(0,0) | 0(0,0) | 0(0,0) |
| Female | 0(0,0) | 0(0,0) | 0(0,0) | 0(0,0) | 0(0,0) | 0(0,0) | 0(0,0.003) | 0(0,0) | 0(0,0.004) | 0(0,0) | 0(0,0) | 0(0,0) | 0(0,0) | 0(0,0) |
| Male | 0(0,0) | 0(0,0) | 0(0,0) | 0(0,0) | 0(0,0) | 0(0,0) | 0(0,0.003) | 0(0,0) | 0(0,0.004) | 0(0,0) | 0(0,0) | 0(0,0) | 0(0,0) | 0(0,0) |
| ***Cognitive processes*** | | | | | | | | | | | | | | |
| Cognitive processes | 0(0,0) | 0(0,0) | 0(0,0) | 0(0,0) | 0(0,0) | 0(0,0) | 0.021(0, 0.028) | 0.021(0, 0.029) | 0.025(0, 0.03) | 0.005(0,0.027) | 0(0,0.023) | 0(0,0) | 0(0,0) | 0(0,0) |
| Insight | 0(0,0) | 0(0,0) | 0(0,0) | 0(0,0) | 0(0,0) | 0(0,0) | 0.004(0, 0.006) | 0.003(0, 0.006) | 0.005(0, 0.007) | 0(0,0.006) | 0(0,0.003) | 0(0,0) | 0(0,0) | 0(0,0) |
| Cause | 0(0,0) | 0(0,0) | 0(0,0) | 0(0,0) | 0(0,0) | 0(0,0) | 0.002(0, 0.004) | 0.001(0, 0.004) | 0.003(0, 0.004) | 0(0,0.003) | 0(0,0.002) | 0(0,0) | 0(0,0) | 0(0,0) |
| Discrepancy | 0(0,0) | 0(0,0) | 0(0,0) | 0(0,0) | 0(0,0) | 0(0,0) | 0.002(0, 0.005) | 0.002(0, 0.005) | 0.004(0, 0.006) | 0(0,0.004) | 0(0,0.003) | 0(0,0) | 0(0,0) | 0(0,0) |
| Tentativeness | 0(0,0) | 0(0,0) | 0(0,0) | 0(0,0) | 0(0,0) | 0(0,0) | 0.004(0, 0.006) | 0.003(0, 0.006) | 0.005(0, 0.006) | 0(0,0.005) | 0(0,0.003) | 0(0,0) | 0(0,0) | 0(0,0) |
| Certainty | 0(0,0) | 0(0,0) | 0(0,0) | 0(0,0) | 0(0,0) | 0(0,0) | 0.003(0, 0.004) | 0.001(0, 0.004) | 0.003(0, 0.005) | 0(0,0.004) | 0(0,0.003) | 0(0,0) | 0(0,0) | 0(0,0) |
| Differentiation | 0(0,0) | 0(0,0) | 0(0,0) | 0(0,0) | 0(0,0) | 0(0,0) | 0.004(0, 0.007) | 0.003(0, 0.007) | 0.005(0, 0.007) | 0(0,0.006) | 0(0,0.004) | 0(0,0) | 0(0,0) | 0(0,0) |
| ***Perceptual processes*** | | | | | | | | | | | | | | |
| Perceptual processes | 0(0,0) | 0(0,0) | 0(0,0) | 0(0,0) | 0(0,0) | 0(0,0) | 0.003(0, 0.005) | 0.002(0, 0.005) | 0.004(0, 0.005) | 0(0,0.004) | 0(0,0.003) | 0(0,0) | 0(0,0) | 0(0,0) |
| See | 0(0,0) | 0(0,0) | 0(0,0) | 0(0,0) | 0(0,0) | 0(0,0) | 0.001(0, 0.005) | 0(0,0.004) | 0.003(0, 0.005) | 0(0,0.002) | 0(0,0) | 0(0,0) | 0(0,0) | 0(0,0) |
| Hear | 0(0,0) | 0(0,0) | 0(0,0) | 0(0,0) | 0(0,0) | 0(0,0) | 0.001(0, 0.004) | 0(0,0.003) | 0.002(0, 0.005) | 0(0,0) | 0(0,0) | 0(0,0) | 0(0,0) | 0(0,0) |
| Feel | 0(0,0) | 0(0,0) | 0(0,0) | 0(0,0) | 0(0,0) | 0(0,0) | 0.005(0, 0.012) | 0(0,0.013) | 0.008(0, 0.015) | 0(0,0.01) | 0(0,0.004) | 0(0,0) | 0(0,0) | 0(0,0) |
| ***Biological processes*** | | | | | | | | | | | | | | |
| Biological processes | 0(0,0) | 0(0,0) | 0(0,0) | 0(0,0) | 0(0,0) | 0(0,0) | 0.022(0, 0.035) | 0.016(0, 0.034) | 0.027(0, 0.038) | 0(0,0.03) | 0(0,0.024) | 0(0,0) | 0(0,0) | 0(0,0) |
| Body | 0(0,0) | 0(0,0) | 0(0,0) | 0(0,0) | 0(0,0) | 0(0,0) | 0.003(0, 0.009) | 0(0,0.007) | 0.005(0, 0.01) | 0(0,0.004) | 0(0,0) | 0(0,0) | 0(0,0) | 0(0,0) |
| Health | 0(0,0) | 0(0,0) | 0(0,0) | 0(0,0) | 0(0,0) | 0(0,0) | 0.01(0, 0.02) | 0.006(0, 0.019) | 0.013(0, 0.02) | 0(0,0.016) | 0(0,0.008) | 0(0,0) | 0(0,0) | 0(0,0) |
| Sexual | 0(0,0) | 0(0,0) | 0(0,0) | 0(0,0) | 0(0,0) | 0(0,0) | 0(0,0.004) | 0(0,0.001) | 0.002(0, 0.005) | 0(0,0) | 0(0,0) | 0(0,0) | 0(0,0) | 0(0,0) |
| Ingest | 0(0,0) | 0(0,0) | 0(0,0) | 0(0,0) | 0(0,0) | 0(0,0) | 0(0,0.002) | 0(0,0) | 0(0,0.002) | 0(0,0) | 0(0,0) | 0(0,0) | 0(0,0) | 0(0,0) |
| ***Drives*** | | | | | | | | | | | | | | |
| Drives | 0(0,0) | 0(0,0) | 0(0,0) | 0(0,0) | 0(0,0) | 0(0,0) | 0.046(0, 0.061) | 0.041(0, 0.065) | 0.053(0, 0.064) | 0(0,0.059) | 0(0,0.045) | 0(0,0) | 0(0,0) | 0(0,0) |
| Affiliation | 0(0,0) | 0(0,0) | 0(0,0) | 0(0,0) | 0(0,0) | 0(0,0) | 0.006(0, 0.013) | 0(0,0.016) | 0.009(0, 0.015) | 0(0,0.012) | 0(0,0) | 0(0,0) | 0(0,0) | 0(0,0) |
| Achievement | 0(0,0) | 0(0,0) | 0(0,0) | 0(0,0) | 0(0,0) | 0(0,0) | 0.008(0, 0.015) | 0.004(0, 0.016) | 0.011(0, 0.017) | 0(0,0.013) | 0(0,0.007) | 0(0,0) | 0(0,0) | 0(0,0) |
| Power | 0(0,0) | 0(0,0) | 0(0,0) | 0(0,0) | 0(0,0) | 0(0,0) | 0.011(0, 0.018) | 0.007(0, 0.019) | 0.014(0, 0.019) | 0(0,0.016) | 0(0,0.008) | 0(0,0) | 0(0,0) | 0(0,0) |
| Reward | 0(0,0) | 0(0,0) | 0(0,0) | 0(0,0) | 0(0,0) | 0(0,0) | 0.009(0, 0.015) | 0.004(0, 0.015) | 0.011(0, 0.016) | 0(0,0.013) | 0(0,0.006) | 0(0,0) | 0(0,0) | 0(0,0) |
| Risk | 0(0,0) | 0(0,0) | 0(0,0) | 0(0,0) | 0(0,0) | 0(0,0) | 0.004(0, 0.008) | 0(0,0.008) | 0.005(0, 0.009) | 0(0,0.006) | 0(0,0) | 0(0,0) | 0(0,0) | 0(0,0) |
| ***Relativity*** | | | | | | | | | | | | | | |
| Relativity | 0(0,0) | 0(0,0) | 0(0,0) | 0(0,0) | 0(0,0) | 0(0,0) | 0.09(0, 0.118) | 0.086(0, 0.123) | 0.104(0, 0.122) | 0(0,0.115) | 0(0,0.098) | 0(0,0) | 0(0,0) | 0(0,0) |
| Motion | 0(0,0) | 0(0,0) | 0(0,0) | 0(0,0) | 0(0,0) | 0(0,0) | 0.01(0, 0.017) | 0.005(0, 0.017) | 0.013(0, 0.019) | 0(0,0.014) | 0(0,0.011) | 0(0,0) | 0(0,0) | 0(0,0) |
| Space | 0(0,0) | 0(0,0) | 0(0,0) | 0(0,0) | 0(0,0) | 0(0,0) | 0.035(0, 0.049) | 0.031(0, 0.052) | 0.04(0, 0.05) | 0(0,0.046) | 0(0,0.037) | 0(0,0) | 0(0,0) | 0(0,0) |
| Time | 0(0,0) | 0(0,0) | 0(0,0) | 0(0,0) | 0(0,0) | 0(0,0) | 0.038(0, 0.054) | 0.033(0, 0.057) | 0.045(0, 0.058) | 0(0,0.052) | 0(0,0.039) | 0(0,0) | 0(0,0) | 0(0,0) |
| ***Time orientations*** | | | | | | | | | | | | | | |
| Past focus | 0(0,0) | 0(0,0) | 0(0,0) | 0(0,0) | 0(0,0) | 0(0,0) | 0.017(0, 0.031) | 0.013(0, 0.034) | 0.024(0, 0.035) | 0(0,0.03) | 0(0,0.016) | 0(0,0) | 0(0,0) | 0(0,0) |
| Present focus | 0(0,0) | 0(0,0) | 0(0,0) | 0(0,0) | 0(0,0) | 0(0,0) | 0.101(0, 0,125) | 0.091(0, 0.127) | 0.111(0, 0.132) | 0.034(0, 0.117) | 0(0,0.103) | 0(0,0) | 0(0,0) | 0(0,0) |
| Future focus | 0(0,0) | 0(0,0) | 0(0,0) | 0(0,0) | 0(0,0) | 0(0,0) | 0.009(0, 0.018) | 0.003(0, 0.015) | 0.012(0, 0.019) | 0(0,0.013) | 0(0,0.011) | 0(0,0) | 0(0,0) | 0(0,0) |
| ***Personal concerns*** | | | | | | | | | | | | | | |
| Work | 0(0,0) | 0(0,0) | 0(0,0) | 0(0,0) | 0(0,0) | 0(0,0) | 0.003(0, 0.01) | 0(0,0.01) | 0.005(0, 0.011) | 0(0,0.007) | 0(0,0) | 0(0,0) | 0(0,0) | 0(0,0) |
| Leisure | 0(0,0) | 0(0,0) | 0(0,0) | 0(0,0) | 0(0,0) | 0(0,0) | 0.001(0, 0.005) | 0(0,0.005) | 0.003(0, 0.006) | 0(0,0.002) | 0(0,0) | 0(0,0) | 0(0,0) | 0(0,0) |
| Home | 0(0,0) | 0(0,0) | 0(0,0) | 0(0,0) | 0(0,0) | 0(0,0) | 0(0,0.003) | 0(0,0.003) | 0.001(0, 0.004) | 0(0,0) | 0(0,0) | 0(0,0) | 0(0,0) | 0(0,0) |
| Money | 0(0,0) | 0(0,0) | 0(0,0) | 0(0,0) | 0(0,0) | 0(0,0) | 0(0,0.002) | 0(0,0.001) | 0.001(0, 0.003) | 0(0,0) | 0(0,0) | 0(0,0) | 0(0,0) | 0(0,0) |
| Religion | 0(0,0) | 0(0,0) | 0(0,0) | 0(0,0) | 0(0,0) | 0(0,0) | 0(0,0.001) | 0(0,0) | 0(0,0.001) | 0(0,0) | 0(0,0) | 0(0,0) | 0(0,0) | 0(0,0) |
| Death | 0(0,0) | 0(0,0) | 0(0,0) | 0(0,0) | 0(0,0) | 0(0,0) | 0.005(0, 0.014) | 0(0,0.012) | 0.009(0, 0.015) | 0(0,0.009) | 0(0,0.005) | 0(0,0) | 0(0,0) | 0(0,0) |
