# Supplementary material for: Identifying Reddit Users at a High Risk of Suicide and Their Linguistic Features During the COVID-19 Pandemic: Growth-Based Trajectory Model
Source: J Med Internet Res. 2024 Aug 8;26:e48907. doi: 10.2196/48907 (PMC11342008; doi:10.2196/48907)
Supplement: Multimedia Appendix 3 [file jmir_v26i1e48907_app3.docx]

**Multimedia Appendix 2.** Trends of LIWC frequency by category for r/SuicideWatch users in the high- and low-risk groups throughout the COVID-19 pandemic (based on the median).

*Appendix 2 continues*

*Appendix 2 continues*
